# Optimal Machine Learning Models for Developing Prognostic Predictions in Patients With Advanced Cancer

**DOI:** 10.7759/cureus.76227

**Published:** 2024-12-22

**Authors:** Jun Hamano, Ayano Takeuchi, Tomoya Keyaki, Hidemasa Nose, Kenichi Hayashi

**Affiliations:** 1 Palliative and Supportive Care, University of Tsukuba, Tsukuba, JPN; 2 Science and Engineering, Chuo University, Tokyo, JPN; 3 Mathematics, Graduate School of Science and Technology, Keio University, Yokohama, JPN; 4 Science and Engineering, Graduate School of Science and Engineering, Chuo University, Tokyo, JPN; 5 Mathematics, Keio University, Yokohama, JPN

**Keywords:** advanced cancer patients, machine learning models, palliative and end-of-life care, prognostic prediction, traditional statistical models

## Abstract

Context: Accurate prognosis prediction for cancer patients in palliative care is critical for clinical decision-making and personalized care. Traditional statistical models have been complemented by machine learning approaches; however, their comparative effectiveness remains underexplored.

Objectives: To assess the prognostic accuracy of statistical and machine learning models in predicting 30-day survival in patients with advanced cancer using objective data, such as the result of the blood test.

Methods: A secondary analysis of the Japan-Prognostic Assessment Tools Validation (J-ProVal) study was performed from September 2012 to April 2014. We used data from 58 palliative care services in Japan and enrolled 915 patients. Four models, fractional polynomial (FP) regression, Kernel Fisher discriminant analysis (KFDA), Kernel support vector machine (KSVM), and XGBoost, were compared using 17 objective clinical characteristics. Models were evaluated with the area under the receiver operating characteristic curve (AUC) as the primary metric.

Results: The KSVM model demonstrated the highest predictive accuracy (AUC: 0.834), outperforming the FP model (AUC: 0.799). XGBoost showed comparatively lower performance; however, it was likely limited by the size of the dataset.

Conclusions: Machine learning, particularly KSVM, has high predictive accuracy in palliative care when sufficient data are available. However, our findings suggest that traditional statistical models offer advantages in stability and interpretability, underscoring the importance of tailored model selection based on data characteristics.

## Introduction

Accurate prognosis prediction is essential for informed clinical decision-making, resource allocation, and patient-centered treatment options and goals of care discussion [1-4]. However, predicting the prognosis of cancer patients is challenging due to a complex interplay of various factors. Physician-based prognostication is often inaccurate, with a tendency to overestimate survival [5].

Prognostic models for cancer patients in palliative care have evolved over the years. Since the 1990s, subjective data, such as physical symptoms, have been incorporated into these models [6]. Recently, there has been a shift toward using objective data, including laboratory test results. Previous studies reported that objective data-based models offer superior performance in palliative care settings [7,8].

Fractional polynomials (FP) modeling is a method that is recommended by the Guidelines for Transparent Reporting of a Multivariable Model for Individual Prognosis or Diagnosis [9]. Our previous study reported that FP modeling had better accuracy than existing prediction models using only objective data in palliative care [7].

Traditional statistical methods, such as FP, have been the primary approach for developing objective data-based prognostic models in palliative care. However, machine learning techniques for developing prediction models have emerged as a promising alternative method in recent years [10,11]. The FP model is an extension of traditional regression in statistics that expresses the relationships between the outcome and the patient characteristics. In this study, we apply three machine learning techniques. Kernel Fisher discriminant analysis (KFDA) and Kernel support vector machines (KSVM) are methods that use Kernel techniques to handle complex, nonlinear patterns in data. XGBoost is a tree-based method that combines multiple decision trees to improve prediction accuracy, offering features like nonlinear prediction and efficient computation. Statistical models enable us to understand relationships within data, emphasizing the evaluation of statistical parameters and their inference by confidence intervals and hypothesis testing. They prioritize interpretability, allowing for clear insights into the influence of each variable. In contrast, machine learning techniques aim to learn patterns from data to enhance prediction and classification accuracy. They often prioritize predictive performance over interpretability, seeking to generalize to future data.

Although there is a burgeoning interest in using machine learning for prognostication, the comparative performance of statistical and machine learning models in palliative care remains to be elucidated. We aimed to compare the prognostic accuracy of statistical and machine-learning models in predicting the survival of cancer patients using objective data in palliative care.

## Materials and methods

We performed a secondary analysis of the Japan-prognostic assessment tools validation (J-ProVal) study. The J-ProVal was a multicenter prospective cohort study that was performed to investigate the feasibility and accuracy of existing prognostic tools. The methodology has been reported in detail elsewhere [12-14].

We compared the prediction performances of various statistical methods or machine learning methods in predicting the prognosis of patients with advanced cancer. Data were obtained from 58 palliative care services in Japan from September 2012 through April 2014. The participating services included 19 hospital palliative care teams, 16 palliative care units, and 23 home-based palliative care services. The primary physician of each patient performed an evaluation and recorded the demographic and clinical characteristics of the participants. Data from laboratory tests were only obtained if the patient underwent blood tests as a clinical necessity within one week after enrolment.

The J-ProVal study was conducted in accordance with the ethical standards of the Declaration of Helsinki and the ethical guidelines for research presented by the Ministry of Health, Labour, and Welfare of Japan. The Institutional Review Boards of all participating services approved this study and the use of the existing J-ProVal data for secondary analysis. The authors did not have access to any information that could identify individual participants during or after data collection.

Patients

Eligible patients were enrolled consecutively as they were referred to the participating services throughout the study period. All services were asked to evaluate and collect data on a specific number of patients, such as 20, 40, 60, 80, or 100, based on the size of the service. Patients were eligible for the study if they were adults (aged 20 years or older); had locally advanced or metastatic cancer (including hematopoietic neoplasms); and had been admitted to a palliative care unit, referred to a hospital palliative care team, or were receiving home-based palliative care.

Measurements and variables

Survival time was defined as the period from the day of referral to the date of death. If patients survived for <6 months after enrollment, survival time was defined as 180 days and they were censored at that time.

The physician assessed variables on the day of admission, including the patient's age, sex, site of the primary tumor and metastases, anticancer treatment during the one-month period before assessment (such as chemotherapy, hormone therapy, or radiotherapy), symptoms and general condition, vital signs (heart rate and respiratory rate), and the results of blood tests performed during the one-week period before assessment.

We also recorded the demographic and clinical characteristics of the participants, including the age, gender, site of primary cancer, metastatic site, Eastern Cooperative Oncology Group performance status, and anticancer therapy (including chemotherapy, hormone therapy, and radiotherapy).

Statistical analysis

We collected the following patient characteristics: heart rate, respiratory rate, leukocyte count ($10^9$/L), neutrophil count ($10^9$/L), lymphocyte count ($10^9$/L), platelet count ($10^9$/L), urea (mg/dL), creatinine (mg/dL), alanine transaminase (U/L), alkaline phosphatase (U/L), total bilirubin (mg/dL), lactate dehydrogenase (U/L), albumin (g/dL), and C-reactive protein (mg/dL). Using patient characteristics as features, we predicted whether patients would survive 30 days after enrollment.

We also included additional features calculated from these 14 patient characteristics: neutrophil to lymphocyte ratio [15], prognostic nutritional index (prognostic indicator derived from serum albumin levels and total lymphocyte count) [16], and platelet to lymphocyte ratio [17]. Thus, we used a total of 17 features in the analysis, which is the same as the analyses conducted in our previous study [7]. Four models were used for prediction: FP regression [18], KFDA[19], KSVM[20], and XGBoost [21].

FP model 

In this study, we apply the logistic regression model as a statistical method. The logistic regression model expresses (the logarithms of) the odds of the outcomes $y$ by a weighted sum such as $\beta_0+\beta_1x_1+\cdots+\beta_dx_d$, where $x_j$s are patient characteristics (explanatory variables). FP model is an extension of the logistic regression model by using the exponent of explanatory variables, hence the odds are expressed by $\beta_0+\beta_1x_1^p_1+\cdots+\beta_dx_d^p_d$. In our analysis, we use the same value of $p_j$s in Hamano et al. (2018). This model is called a linear model even when $x_j^-1$ or $x_j^2$ is used in the context of statistical models because the outcome is expressed by the sum of them (linear combination). While this model is less flexible than the machine learning techniques that will be described below, it has advantages in the sense that we can understand the influence of each variable $x_j$ to $y$ by the magnitude of $\beta_j$, evaluate its stability by the confidence intervals, and test its significance.

KFDA and KSVM 

KFDA and KSVM are commonly used discriminative models. KFDA discriminates classes of observations with a $d$-dimensional feature vector $\boldsymbol{x}$ using the function $f(\boldsymbol{x})=\boldsymbol{a}^\top\boldsymbol{x}$, where a is the d-dimensional coefficient vector. In KFDA, a is determined by maximizing the ratio of the between-group variance to the within-group variance [19].

KSVM is a method that discriminates sets by maximizing the distance (margin) between two parallel hyperplanes $g(\boldsymbol{x})=1$ and $g(\boldsymbol{x})=-1$ that separate the two classes of data. KSVM discriminates classes of observations with the $d$-dimensional feature vector $\boldsymbol{x}$ using the function $g(\boldsymbol{x})=\boldsymbol{w}^\top\boldsymbol{x}-b$, where $\boldsymbol{w}$ is a $d$-dimensional coefficient vector and $b$ is a constant. In KSVM, sets are discriminated such that $y\cdot g(\boldsymbol{x})\geq1$ for a feature $\boldsymbol{x}$ and an objective function $y\in\{-1,1\}$. However, this condition only applies in linearly discriminative cases; thus, the constraints are relaxed and set $y\cdot g(\boldsymbol{x})\geq1-\xi$, where ξ is the slack variable and represents the degree of violation between two hyperplanes $g(\boldsymbol{x})=1$ and $g(\boldsymbol{x})=-1$. In KSVM, the sum of slack variables increases as the margin increases. KSVM discriminates sets by increasing the margin while keeping the sum of slack variables as small as possible [19].

**Figure 1:**
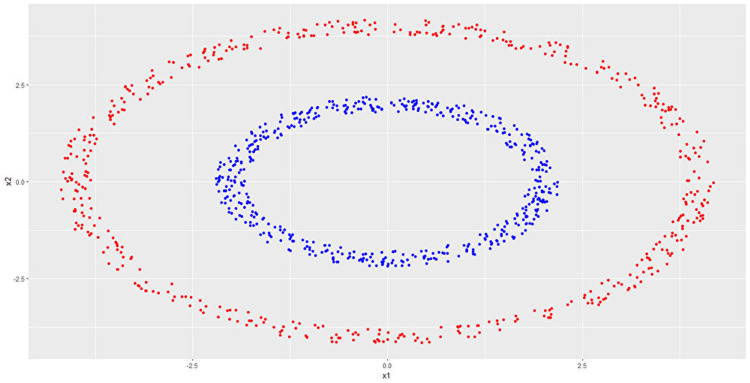
Example of nonlinear transformation (colors indicate groups) Data that cannot be discriminated linearly

**Figure 2:**
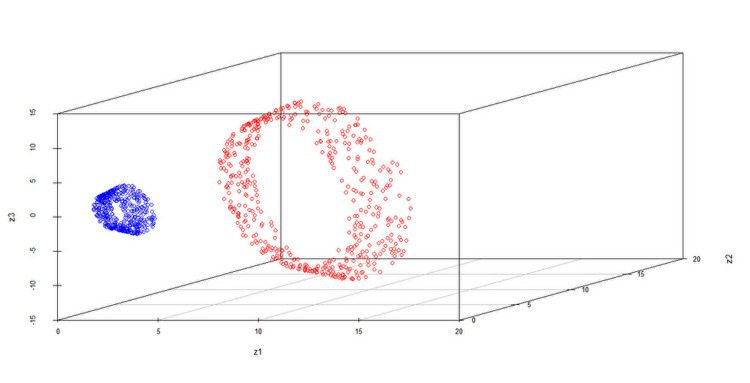
Example of nonlinear transformation (colors indicate groups) Data after transformation to allow for linear discrimination

In practice, linear discriminant methods, such as KFDA and KSVM, sometimes fail to discriminate data. In such cases, discriminant performance can be improved by applying nonlinear transformations to the data. For example, Figure 1 below shows data that were difficult to discriminate linearly, but by applying the transformation $(x_1, x_2)\mapsto(x_1^2, x_2^2,\sqrt{2}x_1x_2)$, linear discrimination became possible (Figure 2).

The kernel method is an efficient method for computing the inner product of data with such nonlinear transformations. Using the kernel method, the inner product of the transformed values $\boldsymbol{\phi}(\boldsymbol{x}_i)$ and $\boldsymbol{\phi}(\boldsymbol{x}_j)$ by a nonlinear function$\boldsymbol{\phi}$ is represented by the kernel function $\langle\boldsymbol{\phi}(\boldsymbol{x}_i),\boldsymbol{\phi}(\boldsymbol{x}_j)\rangle=\kappa(\boldsymbol{x}_i,\boldsymbol{x}_j)$. In many analytical methods, it is not necessary to explicitly express the nonlinear transformation; rather, it is sufficient to perform an inner product calculation [22]. This technique, known as the kernel trick, achieves calculations in complex nonlinear spaces. Thus, KFDA and KSVM are methods that apply kernel methods to KFDA and KSVM, respectively [19,20]. Both of these methods use the kernel method for flexible nonlinear discrimination. On the other hand, they differ in terms of dimensionality compression, robustness against outliers, and computational speed. KFDA performs dimensionality reduction to avoid the "curse of dimensionality," in which accuracy is reduced when the number of features is large. Compared to KFDA, KSVM is less affected by outliers and can reduce the number of calculations by using only part of the data [20,23]. For the purpose of the study, a kernel function called Gaussian kernel $\left(\left(\kappa(\boldsymbol{x}_i,\boldsymbol{x}_j)=\text{exp}\left\{-||\boldsymbol{x}_i-\boldsymbol{x}_j||^2/(2\sigma^2)\right\}\,,\sigma>0\right)\right)$ was used.

XGBoost

One of the methods for discrimination is based on decision trees. A decision tree is a method that repeatedly divides the data according to the conditions related to the feature values and discriminates the observed values by the assigned leaf nodes. While decision trees are easy to interpret, they often exhibit poor generalization ability for unknown data [10]. One solution to this issue is ensemble learning. Among the various ensemble techniques, boosting is a method that improves predictive performance by sequentially adding weak classifiers, thereby reducing the discrepancy between predicted and observed values. For the present study, we used XGBoost, a boosting method using decision trees. XGBoost is characterized by the regularization of the objective function, an efficient split-finding algorithm, the ability to handle sparsity caused by missing values and dummy variables, and fast execution speed. XGBoost has demonstrated excellent results in data analysis [21].

Evaluation 

In the analysis, we used the area under the receiver operating characteristic curve (AUC) for evaluation according to our previous study [7]. To evaluate generalization performance for unknown data, five-fold cross-validation was conducted for four methods, and the test AUC values were compared. Training AUC values were used. For the FP model, the variables and degrees determined in our previous study were used. For statistical analyses, KFDA, kernlab, and XGBoost packages in R were used.

## Results

From the total of 2426 patients, those who had missing values for the 17 features or whose date of death was missing were excluded. Therefore, 915 patients were included in the analysis. Table 1 shows the descriptive statistics of each feature for all patients and for the patients that were included in the analysis. The results of the prediction of 30-day survival for each method are shown in Table 2. Based on the AUC values, the FP model, which is an extension of the polynomial model, outperformed KFDA and XGBoost, which are more flexible methods. On the other hand, the prediction performance of KSVM, which is similar to KFDA in terms of the use of the kernel method, exceeded that of the FP model.

**Table 1:** Patient characteristics

Characteristic	N	%
Age (mean, SD)	68.3±12.9	
Gender		
Male	562	61.4
Site of primary cancer		
Lung	222	24.7
Gastrointestinal	244	27.2
Gynecological	47	5.2
Urogenital	44	4.9
Breast	47	5.2
Other	293	32.7
Metastatic site		
Anywhere	767	84.0
Liver	378	41.5
Bone	256	28.1
Lung	314	34.4
Central nervous system	113	12.4
ECOG performance status		
0-1	63	6.9
2	163	17.9
3	332	36.4
4	355	38.9
Anticancer therapy		
Chemotherapy	191	20.9
Hormone therapy	7	0.8
Radiotherapy	43	4.7

**Table 2:** Predictive accuracy of 30-day survival ROC-AUC, receiver operating characteristic-area under the curve; FP, fractional polynomial; KFDA, Kernel Fisher discriminant analysis; KSVM, Kernel support vector machine

Methods	FP model	KFDA	KSVM	XGBoost
ROC-AUC (95%CI)	0.799 (0.734-0.864)	0.765 (0.696-0.835)	0.834 (0.807-0.861)	0.784 (0.713-0.848)

## Discussion

To the best of our knowledge, this is the first survey to compare the prognostic accuracy of statistical and machine-learning models in predicting the survival of cancer patients using only objective data in palliative care. The most important finding of our study was that KSVM, which is one of the machine learning models, had better prognostic accuracy for 30-day survival among cancer patients in palliative care. The superior predictive ability of KSVM compared to FP may be attributed to the nonlinear nature of the function, which can flexibly define discriminant boundaries even when the data are distributed in a complex manner using the kernel method [24,25]. However, because the confidence intervals largely overlap with those of FP, it is reasonable to consider that FP possesses some predictive capability, despite being a relatively simple statistical model, rather than viewing KSVM as inherently superior. It is important to compare the accuracy of the statistical models, such as FP, and machine learning techniques, such as KSVM, because the performance of predictive accuracy depends on the size of training data and intrinsic characteristics of the data itself [26]. It is expected that machine learning techniques will perform better than statistical methods as the size of the dataset increases; however, when the results of the two are comparable, the latter has beneficial aspects thanks to the stability and interpretability of the results [27-29]. Notably, XGBoost showed inferior predictive ability compared to predictive models developed using statistical methods. One possible interpretation is that the decision tree used in XGBoost may not effectively approximate linear models, whereas the FP model approximates a linear model [30]. Another possible interpretation is that XGBoost does not perform well on small data sets [31-33]; therefore, XGBoost may not be the optimal method for developing prognostic models in palliative care when working with limited data.

In this study, we analyzed the data by dichotomizing survival time as an outcome, thereby placing it in a more tractable supervised learning setting. The development of predictive models with survival time itself as the outcome remains a methodological challenge. Future investigations may explore the use of statistical methods based on the proportional hazards model and its implementation in machine learning techniques [34-36]. Such survival-time response regression models for survival time response often focus on the estimation of hazard ratios, which requires the potentially unrealistic assumption of proportional hazards. Alternative approaches, such as methods that use pseudo-values to directly estimate the survival function, or those that focus on the restricted mean survival time as the primary outcome [37,38], could offer promising solutions. These statistical and machine learning-based techniques are still in development, and their performance on datasets of the size we analyzed needs to be thoroughly validated in future studies.

Our study had several limitations. The first limitation was that this validation study was conducted only in patients who required blood tests in ordinary clinical settings. In the J-ProVal study, 1263 patients (52.1 %) had no laboratory data and were excluded from the present analysis. For this reason, 1106 (46.8%) patients were excluded. The possible reason with no laboratory data could be the physician’s practice policy, the patient’s refusal, and the patients with too serious conditions. A second limitation is that the patients with missing values for the 17 characteristics or missing date of death were excluded. As a result, only 915 patients (37.7%) could be included in the analysis. Therefore, the results of this study must be interpreted with caution because of selection bias. A third limitation is that our study only included Japanese participants, so caution is needed when applying our results to other ethnic groups.

## Conclusions

Our findings suggest that when a large amount of data is available on cancer patients receiving palliative care, a machine learning method, such as KSVM, could be a viable option for developing a prognostic prediction model using only objective data. However, further studies are needed to compare the accuracy of the KSVM and FP models, as the accuracy of KSVM may vary depending on the size of the training data, whereas the FP model demonstrates stable and consistent accuracy.

## References

[1] Hui D, Paiva CE, Del Fabbro EG (2019). Prognostication in advanced cancer: update and directions for future research. Support Care Cancer.

[2] Degner LF, Kristjanson LJ, Bowman D (2017). Information needs and decisional preferences in women with breast cancer. J Am Med Assoc.

[3] Kirk P, Kirk I, Kristjanson LJ (2004). What do patients receiving palliative care for cancer and their families want to be told? A Canadian and Australian qualitative study. BMJ.

[4] Steinhauser KE, Christakis NA, Clipp EC, McNeilly M, McIntyre L, Tulsky JA (2000). Factors considered important at the end of life by patients, family, physicians, and other care providers. J Am Med Assoc.

[5] Glare P, Virik K, Jones M, Hudson M, Eychmuller S, Simes J, Christakis N (2003). A systematic review of physicians' survival predictions in terminally ill cancer patients. BMJ.

[6] Maltoni M, Caraceni A, Brunelli C (2005). Prognostic factors in advanced cancer patients: evidence-based clinical recommendations--a study by the Steering Committee of the European Association for Palliative Care. J Clin Oncol.

[7] Hamano J, Takeuchi A, Yamaguchi T (2018). A combination of routine laboratory findings and vital signs can predict survival of advanced cancer patients without physician evaluation: a fractional polynomial model. Eur J Cancer.

[8] Simmons CP, McMillan DC, McWilliams K (2017). Prognostic tools in patients with advanced cancer: a systematic review. J Pain Symptom Manage.

[9] Collins GS, Reitsma JB, Altman DG, Moons KG (2015). Transparent reporting of a multivariable prediction Model for individual prognosis or diagnosis (Tripod): the Tripod statement. Ann Intern Med.

[10] Zhang B, Shi H, Wang H (2023). Machine learning and AI in cancer prognosis, prediction, and treatment selection: a critical approach. J Multidiscip Healthc.

[11] Kourou K, Exarchos TP, Exarchos KP, Karamouzis MV, Fotiadis DI (2015). Machine learning applications in cancer prognosis and prediction. Comput Struct Biotechnol J.

[12] Baba M, Maeda I, Morita T (2015). Survival prediction for advanced cancer patients in the real world: a comparison of the Palliative Prognostic Score, Delirium-Palliative Prognostic Score, Palliative Prognostic Index and modified Prognosis in Palliative Care Study predictor model. Eur J Cancer.

[13] Baba M, Maeda I, Morita T (2015). Independent validation of the modified prognosis palliative care study predictor models in three palliative care settings. J Pain Symptom Manage.

[14] Hamano J, Morita T, Ozawa T (2015). Validation of the simplified palliative prognostic index using a single item from the communication capacity scale. J Pain Symptom Manage.

[15] Templeton AJ, McNamara MG, Šeruga B (2014). Prognostic role of neutrophil-to-lymphocyte ratio in solid tumors: a systematic review and meta-analysis. J Natl Cancer Inst.

[16] Zhang C, Wang H, Ning Z, Xu L, Zhuang L, Wang P, Meng Z (2016). Prognostic nutritional index serves as a predictive marker of survival and associates with systemic inflammatory response in metastatic intrahepatic cholangiocarcinoma. Onco Targets Ther.

[17] Templeton AJ, Ace O, McNamara MG (2014). Prognostic role of platelet to lymphocyte ratio in solid tumors: a systematic review and meta-analysis. Cancer Epidemiol Biomarkers Prev.

[18] Royston P, Altman DG (1994). Regression using fractional polynomials of continuous covariates: parsimonious parametric modelling. J R Stat Soc Ser C.

[19] Yang J, Frangi AF, Yang JY, Zhang D, Jin Z (2005). KPCA plus LDA: a complete kernel Fisher discriminant framework for feature extraction and recognition. IEEE Trans Pattern Anal Mach Intell.

[20] Soman KP, Loganathan R, Ajay V (2024). Machine Learning with SVM and Other Kernal Methods. https://books.google.com/books/about/Machine_Learning_with_SVM_and_Other_Kern.html?hl=ja&id=-2evxOIz-d4C.

[21] Tianqi Chen, Tong He MB (2024). Xgboost: extreme gradient boosting. R package version 0.4-2.

[22] Hofmann T, Schölkopf B, Smola AJ (2008). Kernel methods in machine learning. Ann Stat.

[23] Jp STA (2007). Dimensionality reduction of multimodal labeled data by local Fisher discriminant analysis. J Mach Learn Res.

[24] Cortes C, Vapnik V, Saitta L (1995). Support-vector networks. Mach Learn.

[25] Hastie T, Tibshirani R, Friedman J (2009). The Elements of Statistical Learning. Springer New York.

[26] Bennett M, Hayes K, Kleczyk EJ, Mehta R (2024). Similarities and differences between machine learning and traditional advanced statistical modeling in healthcare analytics. Arxiv.

[27] Domingos P (2012). A few useful things to know about machine learning. Commun ACM.

[28] Bzdok D, Altman N, Krzywinski M (2018). Statistics versus machine learning. Nat Methods.

[29] Breiman L (2001). Statistical modeling: the two cultures. Statist Sci.

[30] Raymaekers J, Rousseeuw PJ, Verdonck T, Yao R (2024). Fast linear model trees by PILOT. Mach Learn.

[31] Li J, Liu H, Yang Z, Han L (2021). A credit risk model with small sample data based on G-XGBoost. Appl Artif Intell.

[32] Zou M, Jiang WG, Qin QH, Liu YC, Li ML (2022). Optimized XGBoost model with small dataset for predicting relative density of Ti-6Al-4V parts manufactured by selective laser melting. Materials (Basel).

[33] Silvey S, Liu J (2024). Empirical sample size determination for popular classification algorithms in clinical research. medRxiv.

[34] Kleinbaum DG KM (1996). Survival Analysis: A Self-Learning Text (Statistics in the Health Sciences). http://www.uop.edu.pk/ocontents/survival-analysis-self-learning-book.pdf.

[35] Binder H, Schumacher M (2008). Allowing for mandatory covariates in boosting estimation of sparse high-dimensional survival models. BMC Bioinformatics.

[36] Sanz H, Reverter F, Valim C (2020). Enhancing SVM for survival data using local invariances and weighting. BMC Bioinformatics.

[37] Schenk A, Berger M, Schmid M (2024). Pseudo-value regression trees. Lifetime Data Anal.

[38] Tian L, Zhao L, Wei LJ (2014). Predicting the restricted mean event time with the subject's baseline covariates in survival analysis. Biostatistics.

